# Large-scale gate-all-around MoS_2_ transistor array through lossless monolithic 3D integration

**DOI:** 10.1093/nsr/nwaf539

**Published:** 2025-11-27

**Authors:** Chao Chen, Kuanglei Chen, Hang Zhao, Shucao Lu, Jinsen Shang, He Jiang, Li Gao, Xiaoyu He, Shihua Jiang, Zhangyi Chen, Zheng Zhang, Xiankun Zhang, Yue Zhang

**Affiliations:** Academy for Advanced Interdisciplinary Science and Technology, Key Laboratory of Advanced Materials and Devices for Post-Moore Chips Ministry of Education, State Key Laboratory for Advanced Metals and Materials, University of Science and Technology Beijing, Beijing 100083, China; School of Materials Science and Engineering, Beijing Key Laboratory for Advanced Energy Materials and Technologies, University of Science and Technology Beijing, Beijing 100083, China; Academy for Advanced Interdisciplinary Science and Technology, Key Laboratory of Advanced Materials and Devices for Post-Moore Chips Ministry of Education, State Key Laboratory for Advanced Metals and Materials, University of Science and Technology Beijing, Beijing 100083, China; School of Materials Science and Engineering, Beijing Key Laboratory for Advanced Energy Materials and Technologies, University of Science and Technology Beijing, Beijing 100083, China; Academy for Advanced Interdisciplinary Science and Technology, Key Laboratory of Advanced Materials and Devices for Post-Moore Chips Ministry of Education, State Key Laboratory for Advanced Metals and Materials, University of Science and Technology Beijing, Beijing 100083, China; School of Materials Science and Engineering, Beijing Key Laboratory for Advanced Energy Materials and Technologies, University of Science and Technology Beijing, Beijing 100083, China; Academy for Advanced Interdisciplinary Science and Technology, Key Laboratory of Advanced Materials and Devices for Post-Moore Chips Ministry of Education, State Key Laboratory for Advanced Metals and Materials, University of Science and Technology Beijing, Beijing 100083, China; School of Materials Science and Engineering, Beijing Key Laboratory for Advanced Energy Materials and Technologies, University of Science and Technology Beijing, Beijing 100083, China; Academy for Advanced Interdisciplinary Science and Technology, Key Laboratory of Advanced Materials and Devices for Post-Moore Chips Ministry of Education, State Key Laboratory for Advanced Metals and Materials, University of Science and Technology Beijing, Beijing 100083, China; School of Materials Science and Engineering, Beijing Key Laboratory for Advanced Energy Materials and Technologies, University of Science and Technology Beijing, Beijing 100083, China; Academy for Advanced Interdisciplinary Science and Technology, Key Laboratory of Advanced Materials and Devices for Post-Moore Chips Ministry of Education, State Key Laboratory for Advanced Metals and Materials, University of Science and Technology Beijing, Beijing 100083, China; School of Materials Science and Engineering, Beijing Key Laboratory for Advanced Energy Materials and Technologies, University of Science and Technology Beijing, Beijing 100083, China; Academy for Advanced Interdisciplinary Science and Technology, Key Laboratory of Advanced Materials and Devices for Post-Moore Chips Ministry of Education, State Key Laboratory for Advanced Metals and Materials, University of Science and Technology Beijing, Beijing 100083, China; School of Materials Science and Engineering, Beijing Key Laboratory for Advanced Energy Materials and Technologies, University of Science and Technology Beijing, Beijing 100083, China; Academy for Advanced Interdisciplinary Science and Technology, Key Laboratory of Advanced Materials and Devices for Post-Moore Chips Ministry of Education, State Key Laboratory for Advanced Metals and Materials, University of Science and Technology Beijing, Beijing 100083, China; School of Materials Science and Engineering, Beijing Key Laboratory for Advanced Energy Materials and Technologies, University of Science and Technology Beijing, Beijing 100083, China; Academy for Advanced Interdisciplinary Science and Technology, Key Laboratory of Advanced Materials and Devices for Post-Moore Chips Ministry of Education, State Key Laboratory for Advanced Metals and Materials, University of Science and Technology Beijing, Beijing 100083, China; School of Materials Science and Engineering, Beijing Key Laboratory for Advanced Energy Materials and Technologies, University of Science and Technology Beijing, Beijing 100083, China; Academy for Advanced Interdisciplinary Science and Technology, Key Laboratory of Advanced Materials and Devices for Post-Moore Chips Ministry of Education, State Key Laboratory for Advanced Metals and Materials, University of Science and Technology Beijing, Beijing 100083, China; School of Materials Science and Engineering, Beijing Key Laboratory for Advanced Energy Materials and Technologies, University of Science and Technology Beijing, Beijing 100083, China; Academy for Advanced Interdisciplinary Science and Technology, Key Laboratory of Advanced Materials and Devices for Post-Moore Chips Ministry of Education, State Key Laboratory for Advanced Metals and Materials, University of Science and Technology Beijing, Beijing 100083, China; School of Materials Science and Engineering, Beijing Key Laboratory for Advanced Energy Materials and Technologies, University of Science and Technology Beijing, Beijing 100083, China; Academy for Advanced Interdisciplinary Science and Technology, Key Laboratory of Advanced Materials and Devices for Post-Moore Chips Ministry of Education, State Key Laboratory for Advanced Metals and Materials, University of Science and Technology Beijing, Beijing 100083, China; School of Materials Science and Engineering, Beijing Key Laboratory for Advanced Energy Materials and Technologies, University of Science and Technology Beijing, Beijing 100083, China; Academy for Advanced Interdisciplinary Science and Technology, Key Laboratory of Advanced Materials and Devices for Post-Moore Chips Ministry of Education, State Key Laboratory for Advanced Metals and Materials, University of Science and Technology Beijing, Beijing 100083, China; School of Materials Science and Engineering, Beijing Key Laboratory for Advanced Energy Materials and Technologies, University of Science and Technology Beijing, Beijing 100083, China

**Keywords:** lossless monolithic 3D integration, 2D MoS_2_, large scale, ultrahigh current density, gate-all-around, seed layer

## Abstract

Integrating 2D materials into 3D architectures can break through the physical limits of materials in advanced processes. However, challenges such as severe interfacial doping caused by dielectric deposition during vertical stacking processing have led to performance degradation in MoS_2_ 3D gate-all-around (GAA) field-effect transistors (FETs), thereby severely hindering their large-scale integration. Here, we demonstrate a lossless monolithic 3D (M3D) integration process flow enabled by using an interface engineering strategy to achieve the highly uniform large-scale integration of multichannel MoS_2_ GAAFETs with ultrahigh current density. This strategy involves reducing interface states and dielectric doping by forming van der Waals contacts with MoS_2_ and creating hydrophilic surfaces for high-*κ* dielectric deposition via an Sb_2_O_3_ layer, thereby significantly improving the performance of the GAAFETs. The statistics of 112 GAA devices exhibit record-breaking performance, including an average on-state current density of 227 μA/μm with a peak value of >335 μA/μm, and an ideal minimum subthreshold swing approaching 60 mV/dec, all outperforming conventional back-gate transistors and other MoS_2_ 3D FETs. Furthermore, Technology Computer Aided Design simulation confirms that gate-all-around transistors exhibit a 46% reduction in resistance capacitance delay compared with planar structures, further demonstrating enhanced gate control. This work establishes a manufacturing pathway for achieving the interface-doping-free deposition of gate dielectric layers, thereby addressing the performance-degradation issue caused by repeated processing steps in high-density M3D heterogeneous integration.

## Teaser

A lossless 3D integration process addresses interface-doping challenges, enabling high-performance MoS_2_ GAAFETs suitable for large-scale integration.

## INTRODUCTION

Fin field-effect transistors (FinFETs) and gate-all-around (GAA) field-effect transistors (FETs) technology [1], featuring superior design flexibility, enhanced gate control and industrial scalability compatibility, marked the transition of silicon-based transistors from planar to 3D structures. This transformation enabled further scaling of the contacted gate pitch and sustained Moore’s Law at advanced nodes [2–4]. However, constrained by the intrinsic properties of silicon materials, silicon nanosheets present critical challenges in 3D integration, including the rapid decline of electrical performance at an ultra-small thickness due to high surface roughness and severe interface scattering. 2D semiconductors have become key materials to facilitate the continued scaling of transistor dimensions due to their stable electrical properties at the nanoscale and the van der Waals interactions [5–14]. Nevertheless, during repeated stacking and processing, monolayer 2D materials often inevitably suffer from lattice damage and additional doping, making it difficult to retain the intrinsic structure. These issues further lead to problems such as severe current degradation, excessive threshold voltage shift and reduced reliability [15–21]. Monolithic 3D (M3D) integration schemes based on 2D materials have yet to achieve high-uniformity scalable integration, in which the degradation of material performance in multilayer stacking is a critical factor restricting the development of 2D-material-based M3D technology [22].

In the continuous stacking of 3D GAA structures, issues such as surface impurity adsorption residue [23] and the poor crystalline domain orientation of channel materials [24] can lead to performance degradation, significant deviations in key characteristics and reduced reliability of devices after multichannel integration. Among these, the main challenge affecting M3D performance degradation is the deposition of a high-quality high-*κ* dielectric on 2D material surfaces. Low-defect interfaces are critical for gate control in FETs—especially for monolayer 2D nanosheets with only Angstrom-scale thickness—but damage-free processing and freedom from doping interference remain challenges to overcome [25–27]. Various 2D materials have inert surfaces without dangling bonds and traditional atomic layer deposition (ALD) processes often result in nonuniform oxide coverage with rough structures, failing to achieve an ideal van der Waals interface. Specifically, nucleation typically occurs randomly at defects and grain boundaries, leading to nonuniform dielectric thickness, significant charge trapping, nonuniform capacitance and extrinsic carrier scattering [28]. These issues increase the gate leakage current and severely degrade device performance, thereby affecting the integration of upper-layer devices. Additionally, during the ALD process involving multiple cycles of hafnium and oxygen purging, the exposed semiconductor channel is prone to numerous oxygen vacancies that induce electron doping [29–31], thereby causing a severe negative shift in the threshold voltage. Therefore, there is an urgent need to develop new high-*κ* dielectric integration techniques to preserve the intrinsic material structures. This also serves as a prerequisite for the construction of 3D GAA devices [32,33].

**Figure 1.**
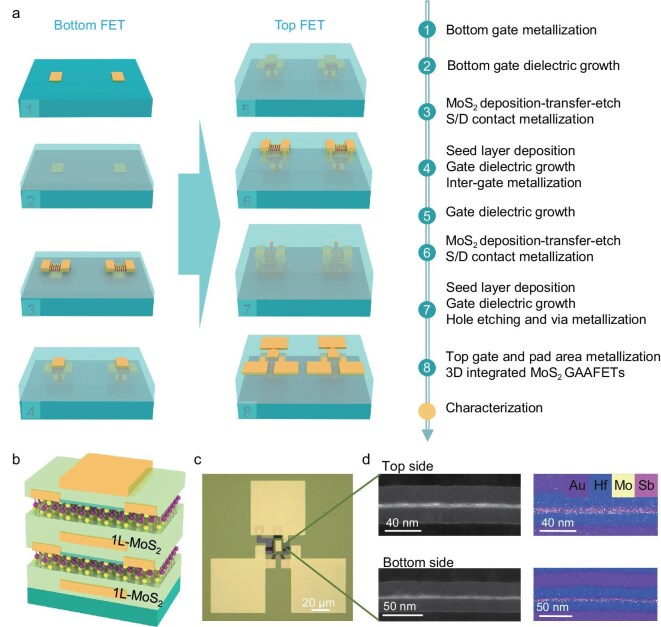
Lossless M3D stacking integration of 2D materials. (a) Lossless M3D integration process flow. (b) 3D schematic of the device structure. (c) Optical microscopy image of the 3D integrated monolayer MoS_2_ GAAFETs. (d) Cross-sectional transmission electron microscopy (TEM) images and energy dispersive spectroscopy mappings of the device.

The mitigating effect of Sb_2_O_3_ on the dependence of ALD-deposition processes on surface dangling bonds has been verified [30]. Nevertheless, there remains a lack of systematic elucidation regarding the role of Sb_2_O_3_ buffer layers in preserving the intrinsic properties of channel materials, particularly during 3D stacking processes. Therefore, we have integrated seed-layer-based high-*κ* dielectric deposition technology into the 3D integration scheme for the first time, achieving a two-step high-*κ* dielectric deposition approach with excellent technical compatibility in multiple processes. Specifically, an ultra-thin inorganic molecular crystal Sb_2_O_3_ seed layer is pre-deposited on the surface of single-crystal monolayer MoS_2_ nanosheets via physical vapor deposition (PVD) to form a van der Waals interface and effectively prevent the damage to channel materials caused by ALD in traditional 3D integration processes. Moreover, the seed layer provides a high-quality hydrophilic surface, enabling the uniform deposition of HfO_2_ high-κ dielectric layers. In combination with silicon-based compatible technologies such as low-temperature damage-free transfer and plasma etching for multiple stacking integrations, the lossless M3D integration process flow of the 2D material is presented in Fig. 1a and the 3D structure of a two-channel MoS_2_ gate-all-around transistor (GAAFET) device is designed and constructed as shown in Fig. 1b and c. Figure 1d indicates that the clean MoS_2_–oxide interfaces are stably maintained under the M3D processing, showcasing remarkable interfacial stability. The fabricated 114 two-channel monolayer MoS_2_ GAAFETs exhibit excellent switching performance and the best uniformity of 98.3% reported to date. Our lossless MoS_2_ stacking scheme can be continuously extended to more channels and the heterogeneous integration of different materials, providing an industry-compatible technological pathway for future 3D-integrated multifunctional logic circuits and 3D heterogeneous chips.

## RESULTS AND DISCUSSION

### Lossless dielectric integration for monolayer MoS_2_ GAAFETs

**Figure 2.**
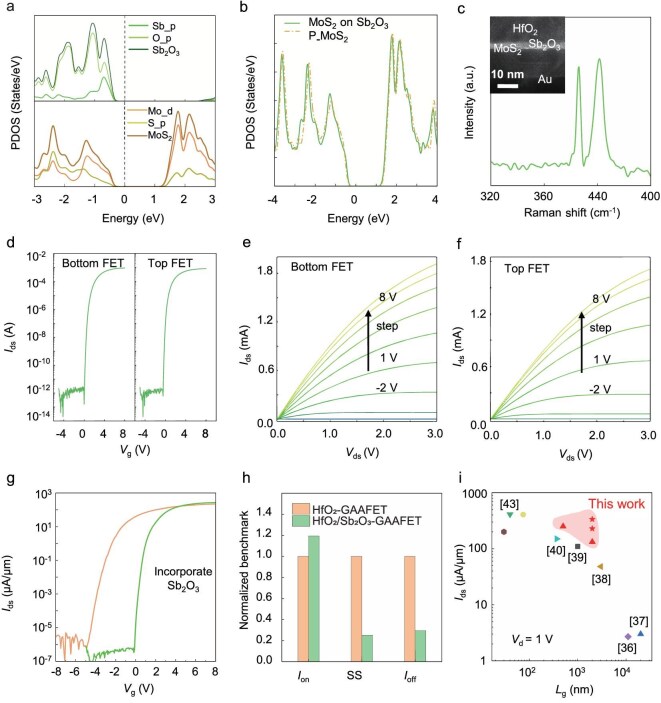
Damage-free interface and performance characterization of 3D integrated GAAFETs. (a) Total density of states (DOS) and the projected DOS of MoS_2_/Sb_2_O_3_. (b) DOS of monolayer MoS_2_ on Sb_2_O_3_ (111) and pristine monolayer MoS_2_. (c) Raman characterization of monolayer MoS_2_ thin films covered with Sb_2_O_3_/HfO_2_ (3/18 nm); inset: TEM image of the gate dielectric–channel interface. (d) Transfer characteristics of the upper and lower channels of MoS_2_ GAAFETs controlled by the same intermediate gate. The left panel shows the lower device, while the right panel shows the upper device, *V*_ds_: 1 V. (e) Output characteristics of the lower channel of MoS_2_ GAAFETs, with the drain voltage scanned from –2 to 8 V, step: 1 V. (f) Output characteristics of the upper channel of MoS_2_ GAAFETs, with the drain voltage scanned from –2 to 8 V, step: 1 V. (g) Comparison of transfer characteristic curves between seedless GAAFET and same-specification GAAFET fabricated via the lossless M3D integration process flow with the assistance of Sb_2_O_3_ seed layer (channel width:length ratio = 2μm:8 μm, *V*_ds_: 1 V). (h) Normalized comparison of critical performance metrics between the two GAAFETs. (i) Performance benchmarking of 3D GAAFETs. This plot shows the maximum on-current density achieved in various reported devices versus their corresponding gate length. The devices corresponding to each data point are as follows: two-channel 1/2L-MoS_2_ GAAFET, exfoliation [36]; two-channel 3L-MoS_2_ multi-bridge channel field-effect transistors, chemical vapor deposition (CVD) [37]; three-channel 1L-MoS_2_ GAAFET, CVD [38]; two-channel 1L-MoS_2_ GAAFET, CVD [39]; two-channel ML-MoS_2_ GAAFET, exfoliation [40]; one-channel 1L-MoS_2_ GAAFET-Taiwan Semiconductor Manufacturing Company, sub-atmospheric chemical vapor deposition [41]; ML-MoS_2_ FinFET, CVD [42]; two-channel Si GAAFET, epitaxy [43]; one-channel 1L-MoS_2_ GAAFET (triangle) and two-channel 1L-MoS_2_ GAAFET (star), CVD.

Recently, inorganic molecular crystals (IMCs) have emerged as a novel class of high-*κ* dielectrics, composed of self-passivated molecular clusters connected via van der Waals forces, thus enabling their application in direct deposition on 2D semiconductors to form high-quality dielectric layers. In this work, we investigate the use of Sb_2_O_3_ as a seed layer to assist in the ALD of HfO_2_ dielectric materials, aiming to achieve a lossless top-gate dielectric–channel interface during repeated 3D stacking processes. We employ first-principles calculations to systematically study the interfacial interactions and electronic structures at the Sb_2_O_3_/monolayer MoS_2_ interface, providing a theoretical foundation for subsequent experimental investigations. The calculated adsorption energy (*E*_ad_) of Sb_2_O_3_/MoS_2_ is –22.80 meV/Å^2^, which is significantly lower than the adsorption energies of interfaces such as HfO_2_/MoS_2_ [34], verifying that the dominant interaction at this interface is van der Waals forces. Subsequently, as shown in Fig. 2a, we present the projected density of states (PDOS) for the intrinsic monolayer MoS_2_, Sb_2_O_3_ material. Analysis reveals that the electron states at the conduction-band edge are primarily contributed by the d orbitals of Mo, while the valence-band edge is dominated by the hybridization of Mo d orbitals and S p orbitals. The electronic contributions of Sb_2_O_3_ are located in deep energy levels far from the Fermi level. In Fig. 2b, we display the PDOS of intrinsic MoS_2_ and MoS_2_ after Sb_2_O_3_ deposition. No obvious new peaks appear in the density of states of interfacial MoS_2_ near the Fermi level after the introduction of Sb_2_O_3_, indicating no introduction of interfacial states or defect states, meaning that the electronic structure of the interface remains fundamentally intact. Subsequently, the band-offset situation at the interface based on Anderson’s rule indicates that this interface exhibits a strongly asymmetric type-I band alignment. The large conduction-band offset (1.09 eV) is beneficial for suppressing electron tunneling and can effectively reduce the risk of gate current leakage. The above calculation results provide solid and reliable theoretical support for the design of subsequent experiments and the analysis of experimental results.

To address the challenges of integrating high-*κ* dielectrics on 2D material surfaces, we propose a silicon-compatible 3D integration flow for 2D materials in which top-gate dielectric integration is achieved based on the seed-layer-assisted deposition strategy. The large-area, high-quality monolayer MoS_2_ prepared on the surface of a fused glass substrate by using chemical vapor deposition and low-temperature, damage-free transfer techniques, as show in Figs S1–S3, possesses large single-crystal materials with low-roughness (*R*_a_ < 0.27 nm) flat surfaces before stacking processing [35]. Under an ultrahigh vacuum (∼10^−8^ Torr), IMCs of Sb_2_O_3_ were thermally evaporated onto 2D MoS_2_ films via PVD, forming a uniform buffer layer. By precisely controlling the evaporation rate at <0.1 Å/s, we achieved a 3-nm-thick continuous film that completely covered the intrinsically hydrophobic MoS_2_ surface. The stronger surface adsorption energy of Sb_2_O_3_ for H_2_O can promote the adsorption of precursors during the ALD process. As shown in Fig. S1c, this interface demonstrated exceptional hydrophilicity, significantly enhancing compatibility with conventional ALD processes for the seedless integration of ultra-thin high-*κ* dielectrics. Raman and Photoluminescence (PL) spectroscopy characterizations were performed on the intrinsic monolayer MoS_2_ and the same monolayer MoS_2_ uniformly coated with thin-layer HfO_2_, thin-layer Sb_2_O_3_ and thin-layer Sb_2_O_3_/HfO_2_, as shown in Fig. S4 and Fig. 2c; the TEM image of the oxide–MoS_2_–oxide interface is shown in the inset, demonstrating a clean line. The oxygen vacancy doping during the direct ALD HfO_2_ process causes an increase in the electron concentration, leading to a decrease in the out-of-plane vibration frequency, enhanced carrier–phonon coupling resulting in phonon mode softening and the generation of negatively charged tritons from excitons. This is manifested in the Raman spectrum as a significant redshift of ∼2.54 cm^−1^ at the A_1 g_ peak position, reduced intensity and broadened full width at half maximum. Meanwhile, the A peak in the PL spectrum also shows an obvious redshift, partial energy quenching and broadening. After the introduction of Sb_2_O_3_, the peak positions remain basically unchanged, confirming the significant inhibitory effect of Sb_2_O_3_ on interfacial doping. Additionally, the capacitance density of the seed-layer-assisted dielectric layer is measured by using a parallel-plate capacitor to be ∼1.2 μF/cm^2^. The theoretical calculation shows that the interface has an interface defect density of 4.25 × 10^12^ cm^−2^eV^−1^. This basically proves the low damage to the channel caused by the top-gate processing technology. All of the above ensure that 2D MoS_2_ remains structurally intact and free from damage during the fabrication process.

As shown in Fig. 2d, we tested the transfer characteristic curves of two-channel monolayer MoS_2_ GAAFETs constructed via the seeding strategy under the same gate of the GAA structure. Both the top and bottom devices exhibit excellent uniformity, with an on/off ratio reaching nine orders of magnitude, and their output curves show similar electrical behaviors. The maximum current densities of the bottom and top FETs are 115.5 and 104.6 μA/μm, respectively, as shown in Fig. 2e and f, which are basically at the same level. The subthreshold swing (SS) of the top FET reaches 94.8 mV/dec, while that of the bottom FET under the same gate condition is as low as 83.3 mV/dec. This slight difference between the two falls within an acceptable normal error range. Therefore, the nondegraded electrical test results demonstrate that the top-channel material remains undamaged after processing through the lossless M3D integration flow. Additionally, Fig. S5 shows the summarized results of the transfer characteristic curves of the array devices of the top-layer and bottom-layer transistors from the same batch under the control of the same gate, as well as the low hysteresis voltage and extremely low gate-drain current. Those results demonstrate high uniformity, with no performance degradation in the top devices. This confirms the high stability and scalability of our silicon-compatible lossless M3D-integration-process flow. Processed through the technical approach proposed in this study, the 1L-MoS_2_ channel can maintain stable electrical performance after multilayer stacking, thereby facilitating the continuous expansion of higher-layer stacking in the vertical direction.

Furthermore, we fabricated identical two-channel GAAFETs by using both the seedless and the seeding strategies. Transfer curves, as shown in Fig. 2g, reveal a threshold voltage deviation of >4 V, confirming that Sb_2_O_3_ significantly suppresses n-type doping. As shown in Fig. 2h, we conducted a comparison of their maximum on-current density, SS and off-current. The seeded GAA device exhibits significantly stronger gate control, demonstrated by a 19.4% increase in the on-current, a 70.4% reduction in the off-current and a 74.9% decrease in the SS. These results further validate the remarkable advantages of the Sb_2_O_3_-assisted strategy in enabling strong gate electrostatic control and high-quality interface integration during 3D stacking. Moreover, we also present the systematic electrical data of GAAFETs fabricated via the seedless strategy in Figs S6–S8. While the above results demonstrate high current densities, they still cannot avoid the threshold voltage shift caused by electron doping and the excessive performance deviation between devices renders them without practical application value. In contrast, devices fabricated based on the seed strategy effectively address issues such as threshold voltage drift and significant performance discrepancies. Considering device aspect ratios and other dimensional factors, under the same node, the current density of our GAAFETs significantly surpasses those of other relevant reports in the field [36–43], as shown in Fig. 2i. Among them, while an extremely low off-state current is maintained, the on-state current density even approaches those of similar devices that are tens of nanometers in size, further demonstrating the significant scaling potential of our work under nanoscale processes.

Overall, this Sb_2_O_3_-enabled PVD/ALD hybrid integration scheme exhibits three critical advantages for silicon-compatible manufacturing: (i) the enhanced surface adsorption energy of Sb_2_O_3_ for H_2_O promotes efficient precursor adsorption during ALD, enabling high-quality dielectric growth; (ii) optimized gate control with suppressed dopant fluctuation maintains the device turn-on voltage at within 3.8% variation across wafer-scale integration; (iii) full compatibility with GAA architectures achieves superior electrostatic control while preserving interface quality during 3D stacking. The demonstrated process constitutes a pivotal advancement in bridging 2D material integration with established silicon fabrication paradigms, particularly for next-generation M3D integration challenges in which conventional oxide deposition techniques face fundamental limitations in conformality and interface defect control.

Regarding specific fabrication engineering, as well as the seed-layer-assisted top-gate integration strategy, we have achieved the scaled integration of large-area monolayer MoS_2_ GAA metal-oxide-semiconductor field-effect transistor (MOSFET) arrays. This was accomplished through multiple lithography and electron-beam exposure alignment processes, combined with multiple etching and electrode interconnects following the encapsulation with high-*κ* dielectrics. All devices feature a 5-nm Cr/15-nm Au bottom gate, 20-nm Au for other gate electrodes and a dielectric stack consisting of 3-nm Sb_2_O_3_ and 18-nm HfO_2_, as well as the 2D MoS_2_ channel and contact metal (20-nm Au). In this work, Au, as a metal with high conductivity and excellent stability, has been selected to ensure the normal switching of logic states during subsequent voltage signal scanning and to minimize energy-induced damage during ultrahigh vacuum deposition. Fig.S9 provides an optical micrograph of the key fabrication steps and 3D schematic diagrams of the multilayer 3D stacking devices; it can be clearly observed that each process step maintains good cleanliness. For more detailed information on the fabrication process, please refer to the ‘Methods’ section. The atomic force microscopy characterization of the vias corresponding to each layer after dielectric encapsulation is shown in Fig. S10 and provides information on the etching depth and surface morphology. Note that each device in the top layer is precisely positioned directly above the corresponding device in the bottom layer, controlled by the same fully encapsulating metal gate. The entire fabrication process is conducted within a thermal budget of 180°C, ensuring the ability to add multiple layers without degradation of the underlying layers. This approach holds promise for the realization of three or more layers of stacked MoS_2_ devices and extension to complementary metal oxide semiconductor 3D integration.

### Electrical statistical characterization of monolayer MoS_2_ GAAFET arrays

**Figure 3.**
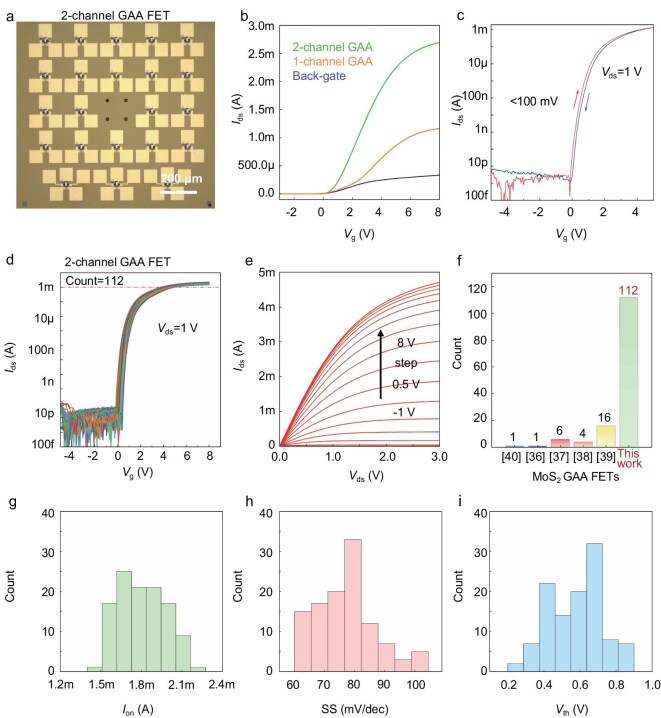
Electrical properties of MoS_2_ GAAFETs. (a) Optical microscopy image of the scaled MoS_2_ GAAFETs. Scale bar: 200 μm. (b) Comparison of transfer characteristics among single-back-gate, dual-gate and GAA devices. (c) Transfer curves of two-channel MoS_2_ GAAFET devices with gate voltage scanned from positive and negative directions, respectively. (d) Transfer characteristics of two-channel MoS_2_ GAAFET arrays constructed by using M3D integration technology under a bias voltage of 1 V, W/L: 8/2 μm. (e) Output characteristics of MoS_2_ GAAFETs, with the gate voltage stepping from –1 to 8 V, step: 0.5 V. (f) Statistics on the integration scale of MoS_2_ GAAFETs constructed in this study and those reported in the literature [36–40]. (g–i) Statistical analysis of the maximum on-current (*I*_on_), SS and threshold voltage (*V*_th_) for the 112 two-channel MoS_2_ GAAFETs, showing normal distribution.

Each fabricated die has an area of 1 cm × 1 cm, with 22 two-channel GAA MOSFETs integrated in each 1 mm × 1 mm region, as shown in Fig. 3a. The total number of devices per die is >550, among which we focused on the electrical transport properties of 114 devices from six randomly selected regions. For low-defect-density materials prepared by using the 2D CZ method, we intentionally extend the gates slightly so as to partially cover the source and drain electrode regions at both ends, thereby avoiding high resistance in the channel region caused by unmodulated gaps beyond the gate control.

Figure 3b presents a comparison of the transfer characteristics of three different gate-controlled structures fabricated in the same batch, all designed at a uniform channel aspect ratio (width:length = 8 μm:2 μm). The two-channel GAA device exhibits a current increase of over four times compared with that of the single-sided modulation of the back-gate device, further demonstrating the excellent current modulation capability of the 3D GAA structure. A comprehensive comparison of several other performance metrics, including current magnitude, SS and on/off ratio, is provided in Fig. S11, and Fig. S1^2^ indicates that the two-channel GAAFETs exhibit superior electrical transport properties and greater robustness in the fabrication process. Figure 3c shows that the hysteresis of the two-channel 1L-MoS_2_ GAAFET between forward and reverse scans is <100 mV, indicating a negligible memory window. This at once demonstrates the high-quality deposition of our dielectric materials and minimal defect trapping at the dielectric/channel interface. Further optimization of the dielectric deposition technique to achieve a lower equivalent oxide thickness and fewer interface defects is expected to continuously reduce the hysteresis window size and enhance the capability of the GAA structure to modulate channel carriers.

Compared with a single-gate structure, the channel control of the GAA structure is significantly more effective, offering potential for future 3D logic circuit construction. As shown by the transfer curves of the two-channel MoS_2_ GAAFETs shown in Fig. 3d, the mean and median values of the on-current density for the array devices are 227.3 and 224.7 μA/μm, respectively, with a standard deviation of 21.6 μA/μm, and the highest extracted on-current density reaches 335 μA/μm. Furthermore, as indicated by the output characteristic curves shown in Fig. 3e, at a drain bias of 3 V, the on-current is >4.8 mA when the gate voltage reaches 8 V, without showing complete saturation. The above current data demonstrate extremely high carrier mobility and fully reflect the excellent carrier modulation capability of the adopted GAA structure. In Fig. 3f, we statistically analysed the device scale of the MoS_2_ GAAFETs vertically stacked in relevant research works and compared them with the number of array devices used in this study for statistics. The significant integration advantage of this study can be observed, further verifying the application potential of the lossless M3D integration process flow.

We conducted a statistical analysis of the key parameters of 112 fabricated devices to further verify the performance uniformity of the 3D devices and the stability of the lossless M3D integration process flow. Figure 3g–i presents histograms of the maximum on-current density, SS and threshold voltage (*V*_th_) for these devices, respectively. Starting from this three core performance indicators, namely the maximum on-current density, SS and *V*_th_, we carried out parameter-extraction and coefficient-of-variation calculations for this array. The results show that the coefficients of variation corresponding to those devices are 9.46%, 12.95% and 4.5%, respectively. These results fully demonstrate that the 3D devices exhibit highly uniform performance characteristics. The on/off-current ratio is >10^10^, demonstrating excellent uniformity and switching characteristics. The mean and median SS values are 76.9 and 77.2 mV/dec, respectively, with a standard deviation of 9.97 mV/dec, and the minimum value approaches the ideal value of 60 mV/dec. These results convincingly demonstrate that high-quality gate oxide dielectric deposition can be achieved based on our proposed scheme and the fabricated two-channel GAAFETs exhibit excellent switching characteristics. The mean and median values of *V*_th_ are 0.58 and 0.59 V, respectively, with a standard deviation of 0.14 V. Those results indicate that *V*_th_ exhibits a small fluctuation range and all transistors are of enhancement mode. In the future, threshold voltage regulation can be achieved through means such as adjusting the device dimensions and electronic doping, which possesses significant scalability potential for 3D logic circuits. These statistical results mark an advanced level among relative research [44]. To explore the extensibility of the seed-layer strategy, we reduced the thickness of the original dielectric layer to 3-nm Sb_2_O_3_ + 9-nm HfO_2_. The electrical properties of the designed and fabricated two-channel monolayer MoS_2_ GAAFET are shown in Fig. S13. The results indicate that, despite a significant reduction in the dielectric-layer thickness, the gate still exhibits stable and efficient channel-control performance. Specifically, under *V*_g_ of 5 V and *V*_ds_ of 1 V, the average current density reaches as high as 157 μA/μm, the average *V*_th_ is maintained at ∼3.9 V and the SS is consistently constrained at <85 mV/dec without significant degradation. These results further demonstrate the application value of the Sb_2_O_3_ seed layer.

### Simulation-assisted 3D integration of 2D materials

**Figure 4.**
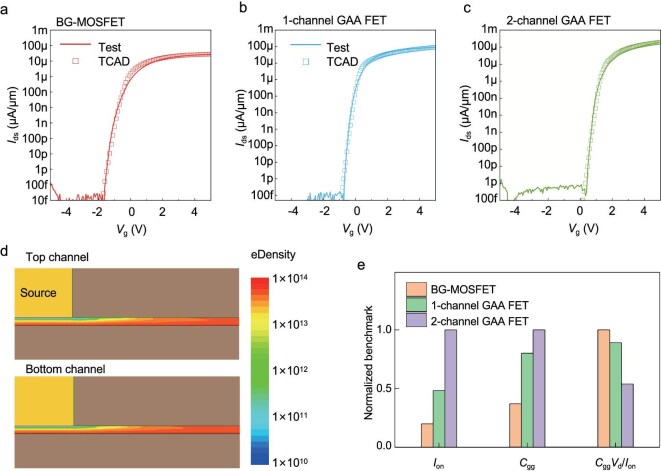
Technology Computer Aided Design (TCAD) simulation of 3D devices. (a–c) Comparison between the TCAD-simulated and experimental transfer characteristics of single-back-gate, dual-gate and GAA devices, *V*_ds_ = 1 V. (d) Distribution of free carrier density in the on-state for the GAA device as simulated by TCAD. (e) Comparison of simulated *I*_on_ values, *C*_gg_ values and gate delays for these devices under *V*_dd_ = 1 V.

To gain deeper insights into the various gate-controlled structures of the MoS_2_ MOSFETs, we conducted detailed Technology Computer Aided Design (TCAD) simulations by using a Sentaurus Device. Figure 4a–c provides a comparison of the simulated and experimental transfer characteristics of the monolayer MoS_2_ transistors with single-back-gate, one-channel GAA and two-channel GAA structures, all of which show high consistency. The ultimate alignment with experimental data indicated that discrepancies in the on-state performance all remained at <10%. Despite the employment of fully back-gated structures during the calibration procedure, strong consistency was achieved when the electrical behaviors of the GAA devices were modeled by using identical parameter sets. This ability to predict outcomes across diverse device structures serves as validation of our model and facilitates the optimization of device and circuit performance. These TCAD models effectively captured the subtle changes in the electrical transport properties of the three different structures, including stronger gate control, as evidenced by a lower SS and higher on/off switching ratios. The TCAD transistor model we developed for the new MoS_2_ material has, to some extent, enabled the development of a process design kit based on 2D material processes. Figure 4d illustrates the distribution of free carrier density in the on-state, showing that the second-layer channel does not exhibit any degradation compared to the first-layer channel, which is in agreement with the experimental results.

Based on the device benchmarking, we derived the on-current (*I*_on_) and total gate capacitance (*C*_gg_) through direct-current and alternating-current simulations under bias conditions of *V*_dd_ = 1 V and *V*_g_ = 5 V, respectively. Subsequently, at the same scaled node, we benchmarked the two-channel GAA structure against two other structures, as depicted in Fig. 4e. The total gate capacitance (*C*_gg_) of the classical back-gated MoS_2_ FET is notably lower. However, the limited gate-control effect leads to a significantly lower *I*_on_ value compared with that of the GAA structure, resulting in a larger final intrinsic gate delay (*C*_gg_*V*_dd_/*I*_on_) than those of the latter two structures. Compared with the back-gated device and the one-channel GAA device, the gate delay of the GAA device with two channels is reduced by 46.15% and 39.58%, respectively. This is because a greater degree of gate wrapping around the channel provides a stronger effect of gate control over carriers, thus maintaining a high *I*_on_ value while incurring a relatively small penalty in terms of *C*_gg_, which fully compensates for the deficiency in the parasitic capacitance. It has been verified that GAA-based 3D devices possess more efficient signal transmission and processing capabilities, concurrently featuring the performance advantages of low power consumption and high energy efficiency. Table S1 provides detailed simulation information.

## CONCLUSION AND PERSPECTIVE

Our work demonstrates a lossless M3D process flow of 2D MoS_2_. Through a two-step high-κ dielectric deposition approach, the seed-layer-assisted modulation improves the vulnerable heterointerfaces in 3D stacking via the van der Waals interface-state-free gap between the 2D MoS_2_ and the dielectric layer, effectively avoiding electron doping of the channel material during fabrication processes. This enables the formation of nearly lossless top devices, with simulation-assisted verification confirming the enhanced gate control and low signal delay of GAA structures. We have achieved the scaled 3D integration of large-area two-channel monolayer MoS_2_ GAAFETs. The 112 enhancement-mode GAAFETs exhibit the best uniformity and electrical performance to date at corresponding technology nodes, with on/off-current ratios of >10^10^, a maximum on-current density reaching 335 μA/μm and a minimum SS as low as 60.26 mV/dec. These results demonstrate the significant performance advantages of 2D MoS_2_ as an n-type material for 3D integration and validate the reliability of the seed-assisted lossless 3D stacking manufacturing process. The finding not only addresses the technical bottlenecks of traditional semiconductor materials in high-density integration, but also offers critical insights for designing and fabricating high-performance 3D electronic devices.

## METHODS

### Large-area integration scheme for 2D MoS_2_ thin films

The large-area single-crystalline MoS_2_ thin films were synthesized by using a novel 2D Czochralski method on the surface of a molten glass substrate. S and MoO_3_ powders are reactant materials and precursors, and float soda-lime glass is the substrate. The other details are outlined in our previous work [35]. Specifically, we utilized an etching reaction to pre-deposit polycrystalline MoS_2_, forming a large-scale 2D Na_2_Mo_2_O_7_ liquid precursor film on the molten glass surface. Subsequently, an ultrafast sulfidation process was employed to obtain large-area MoS_2_ domains on an atomically smooth interface, as shown in Fig. S1a and b. The atomically smooth and defect-free surface of the molten glass substrate was leveraged to enhance the nucleation barriers, thereby reducing the nucleation density during the growth process and weakening the interfacial coupling between the material and the substrate, facilitating subsequent large-scale transfer. Moreover, the high surface tension of the molten glass enabled ultrafast growth rates, making it suitable for large-scale integrated circuit manufacturing.

### Device fabrication and electrical measurements

For the fabrication of the FETs, Cr/Au electrodes are first evaporated onto a SiO_2_/Si substrate. Then, a high-*κ* dielectric layer (HfO_2_, *ε* ∼ 18.9) is deposited by using ALD on the SiO_2_/Si substrate. At this point, the bottom-gate substrate of the FET array is completed. Assisted by water, the MoS_2_ film is transferred onto the patterned substrate. Lithography and O_2_ inductively coupled plasma etch are used to pattern the MoS_2_. Au used as contact electrodes is defined through electron-beam lithography and PVD. Subsequently, the Sb_2_O_3_ is deposited through PVD and the gate dielectric-layer HfO_2_ is deposited. After stacking layer by layer, vias for each layer are formed through SF_6_ and Ar etching, and electrode interconnections are completed through an evaporation process. The devices are annealed in a vacuum at 150°C for 2 hours. Electrical measurements are carried out by using an Agilent B1500A semiconductor tester in a FORMFACTOR^TM^ SUMMIT200 probe station under ambient conditions.

### TCAD simulation

We use Synopsys Sentaurus Device software. Owing to the constraints of commercial software tools, MoS_2_ was equivalently modeled as a bulk semiconductor with a density-functional-theory-calibrated charge model while retaining its actual monolayer thickness, analogous to silicon. The channel-transport dynamics were described via the drift-diffusion approximation. Carrier mobility was characterized by using the Philipp unified model, which accounts for separate limitations from impurity-scattering and phonon-scattering mechanisms. Additionally, mobility degradation due to ionized impurity scattering at the interface was incorporated into the model. Uniformly distributed acceptor traps with consistent energy levels were introduced at MoS_2_-gate oxide interfaces to simulate interfacial weak defects capable of electron capture. Concurrently, donor-trap densities with homogeneous spatial and energetic distributions, based on experimental measurements, were embedded within the MoS_2_ material to represent sulfur vacancies playing the part of electron-carrier sources. Au–MoS_2_ interactions were modeled by using a Schottky contact. Thermally assisted and direct tunneling phenomena at the contact interface were described via a non-local tunneling model employing the Wentzel–Kramers–Brillouin approximation. The ParDiso solver was selected, and the transport equations and Poisson equations were solved self-consistently in a quasi-static scanning manner.

## Supplementary Material

nwaf539_Supplemental_File
