# Supplementary material for: Large-scale gate-all-around MoS2 transistor array through lossless monolithic 3D integration
Source: Natl Sci Rev. 2025 Nov 27;13(6):nwaf539. doi: 10.1093/nsr/nwaf539 (PMC12977324; doi:10.1093/nsr/nwaf539)
Supplement: nwaf539_Supplemental_File [file nwaf539_supplemental_file.pdf]

Supporting Information for

Large scale gate-all-around MoS_2 transistors array by lossless monolithic 3D integration

This PDF file includes:

Supplementary Methods

Fig. S1. First-principles calculation results of Sb_2O_3/MoS_2 interface.

Fig. S2. Schematic illustration of the preparation of monolayer MoS_2 film.

Fig. S3. Low-temperature, damage-free transfer technology of monolayer MoS_2 films.

Fig. S4. Characterization of morphology and structure of monolayer MoS_2 films.

Fig. S5. Spectroscopic characterization of monolayer MoS_2 thin films deposited with different oxide thin films.

Fig. S6. Comparative analysis of 2-channel monolayer MoS_2 GAA FETs.

Fig. S7. Comparison of the electrical transport properties of 3D GAA FETs with variable channel lengths via seedless theory.

Fig. S8. Comparison of electrical transport properties among three different gate control configurations via seedless theory.

Fig. S9. The 2-channel monolayer MoS_2 GAA FET device fabricated via seedless theory.

Fig. S9. Critical fabrication steps during large-scale 3D integration of monolayer MoS_2 GAA FETs.

Fig. S11. AFM topography characterization of vias at different layers of monolayer MoS_2 GAA FET devices.

Fig. S12. Performance statistical analysis in back-gated monolayer MoS_2 MOSFET arrays.

Fig. S13. Performance statistical analysis in 1-channel monolayer MoS_2 GAA FET arrays.

Fig. S14. Performance statistical analysis in 2-channel monolayer MoS_2 GAA FET arrays with thinner gate oxide.

Table S1. The developed material parameter library for MoS_2 and a partial display of the device model parameters constructed based on this library.

Supplementary Methods

Damage-free transfer technology for large-area 2D MoS_2

Owing to the delicate atomic-scale thickness of 2D materials, conventional chemical etching-assisted transfer methods (e.g., HF etching of SiO_2) typically cause significant damage to the material surface (e.g., chemical doping, bubbles, and wrinkles). Additionally, PDMS-based mechanical exfoliation transfer, which relies solely on physical interactions, is limited to small-area single-device studies. Therefore, there is an urgent need to develop a damage-free, large-area, low-temperature transfer technique. Based on the aforementioned large-area 2D MoS_2 material, we utilized the weak interfacial coupling of a smooth substrate surface and combined it with a water-assisted transfer method and the adhesive properties of PMMA. Under the action of capillary forces and the surface tension of deionized water, MoS_2 was spontaneously separated through the gentle insertion of deionized water at the interface. Characterization results revealed that the transferred MoS_2 exhibited nearly ideal surface quality, which has been detailed in our previous work. This damage-free transfer scheme provides a robust technological foundation for the repetitive stacking and large-scale integration of 2D MoS_2 in subsequent applications.

First-principles calculations

We have carried out the DFT calculations by using the Vienna ab-initio simulation package. The Perdew-Burke-Ernzehof (PBE) exchange-correlation functional within the generalized gradient approximation (GGA) approach has been used for DFT simulations. All structures were fully relaxed until the maximum force component acting on each atom was less than 0.02 eV $Å^{-1}$ with an energy convergence criterion of 10^{-5} eV and a cut-off of 450 eV. The $3\times3\times1$ and $5\times5\times1$ k-point meshes were used for geometric optimization and electronic structure calculations, respectively. We set a vacuum thickness of 20 Å to minimize the periodic interactions in the vertical direction. Furthermore, the van der Waals dispersion interactions are incorporated using Grimme's DFT-D3 method.

TCAD simulation

Device simulation models, AI algorithms, and other such tools can effectively promote new materials from laboratory verification to industrial integration. In this article, we use Synopsys Sentaurus Device software to perform the simulation. Owing to constraints of commercial software tools, MoS_2 was equivalently modeled as a bulk semiconductor with density-functional-theory-calibrated charge model while retaining its actual monolayer thickness, analogous to silicon-based materials. The channel transport dynamics were described via the drift-diffusion approximation. Carrier mobility (μ) was characterized using the Philipp unified model, which accounts for separate limitations from impurity scattering and phonon scattering mechanisms. Additionally, mobility degradation due to ionized impurity scattering at the interface was incorporated into the model. Under high lateral electric field conditions, the velocity saturation effect was modeled using the Caughey-Thomas phenomenological approach. Uniformly distributed acceptor traps with consistent energy levels were introduced at MoS_2-gate oxide interfaces to simulate interfacial weak defects capable of electron capture. Concurrently, donor trap densities with homogeneous spatial and energetic distributions, based on experimental measurements, were embedded within the MoS_2 material to represent sulfur vacancies acting as the part of electron carrier sources. Au-MoS_2 interactions were modeled using a Schottky contact. Thermally assisted and direct tunneling phenomena at the contact interface were described via a non-local tunneling model employing the Wentzel-Kramers-Brillouin approximation. The ParDiso solver was selected, and the transport equations and Poisson equations were solved self-consistently in a quasi-static scanning manner.

The ultimate alignment with experimental data indicated that the discrepancy in the on-state performance all remained below 10%. Despite employing fully back-gated structures during the calibration procedure, strong consistency was achieved when modeling the electrical behaviors of GAA devices using identical parameter sets. This ability to predict outcomes across diverse device structures serves as validation for our model and facilitates the optimization of device and circuit performance. For the device benchmarking, we

derived the on-current (I_{on}) and total gate capacitance (C_{gg}) through direct-current and alternating-current simulations under bias conditions of V_{dd} = 1 V and V_g = 5 V, respectively. The gate delay was subsequently computed using the formula $C_{gg}V_{dd}/I_{on}$ based on these obtained parameters.

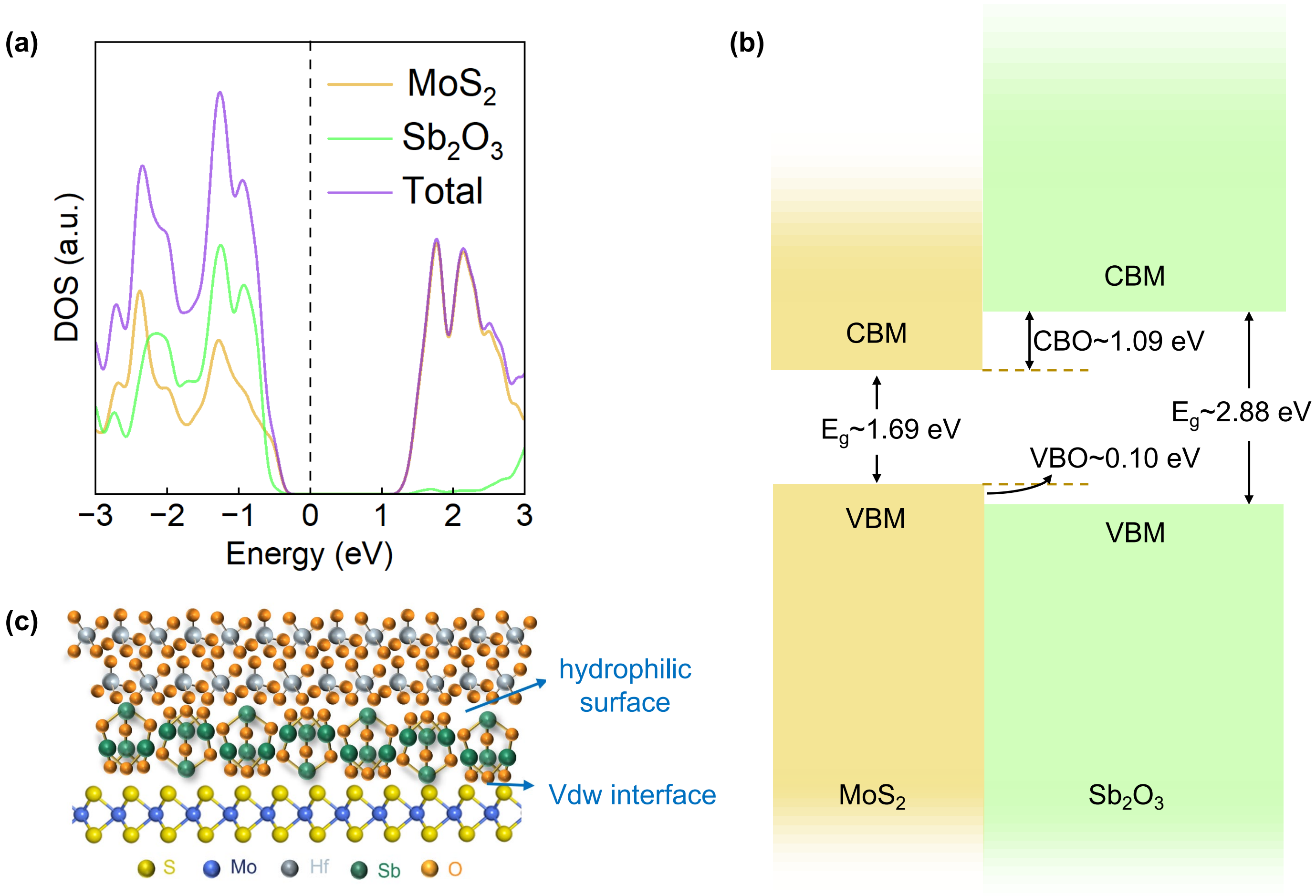

Fig. S1. First-Principles Calculation Results Sb_2O_3/monolayer MoS_2 interface. (a) Total DOS and the projected DOS of MoS_2/Sb_2O_3. (b) Band offset diagram of the MoS_2/Sb_2O_3 interface. (c) Interface structure schematic diagram.

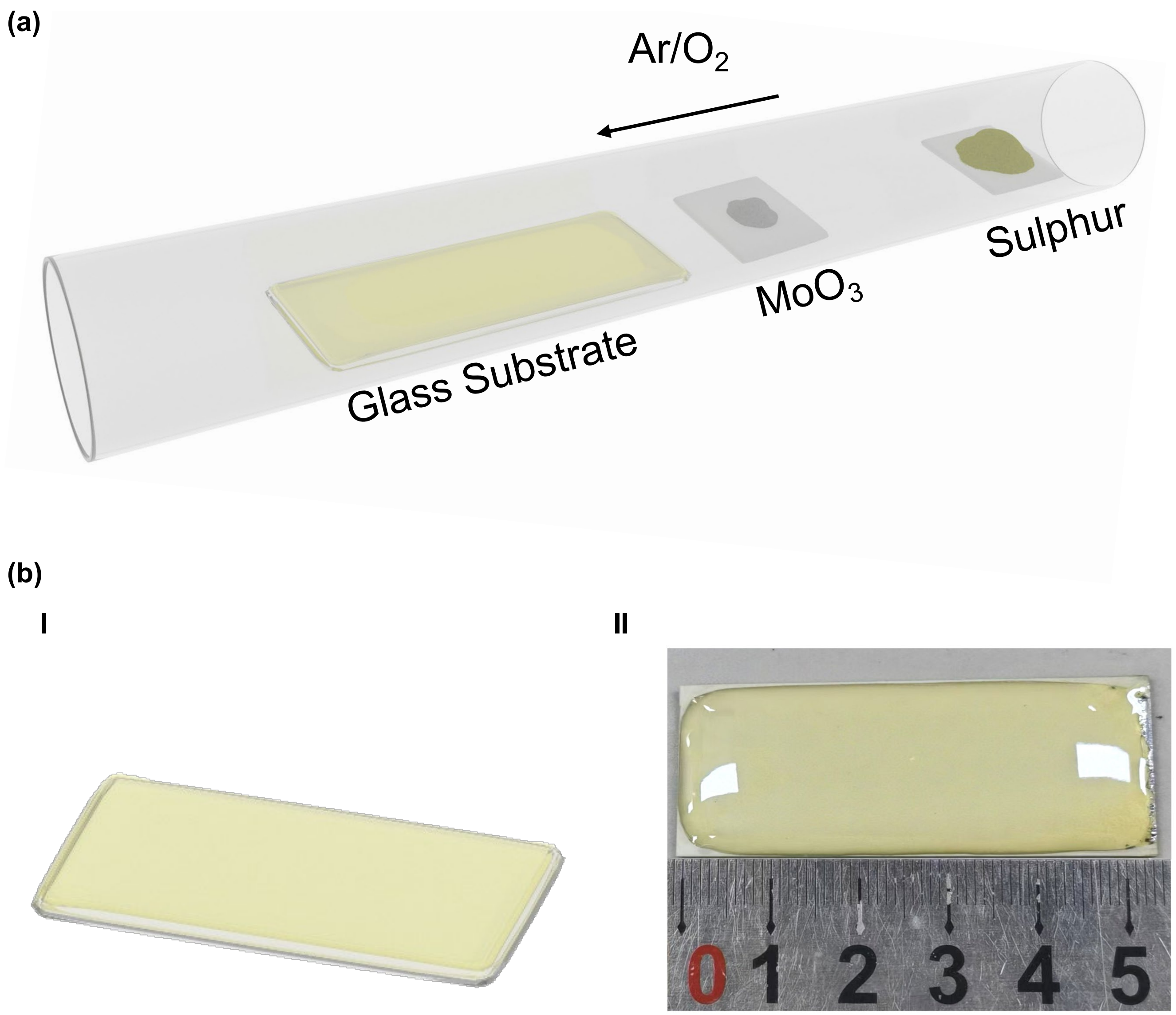

Fig. S2. **Schematic illustration of the preparation of monolayer MoS_2 film.** (a) Schematic illustration of a tube furnace for the 2D Czochralski growth of monolayer MoS_2 films. (b) 3D rendering of a monolayer molybdenum disulfide sample grown on a glass substrate and a photograph of the actual sample (with scale bar).

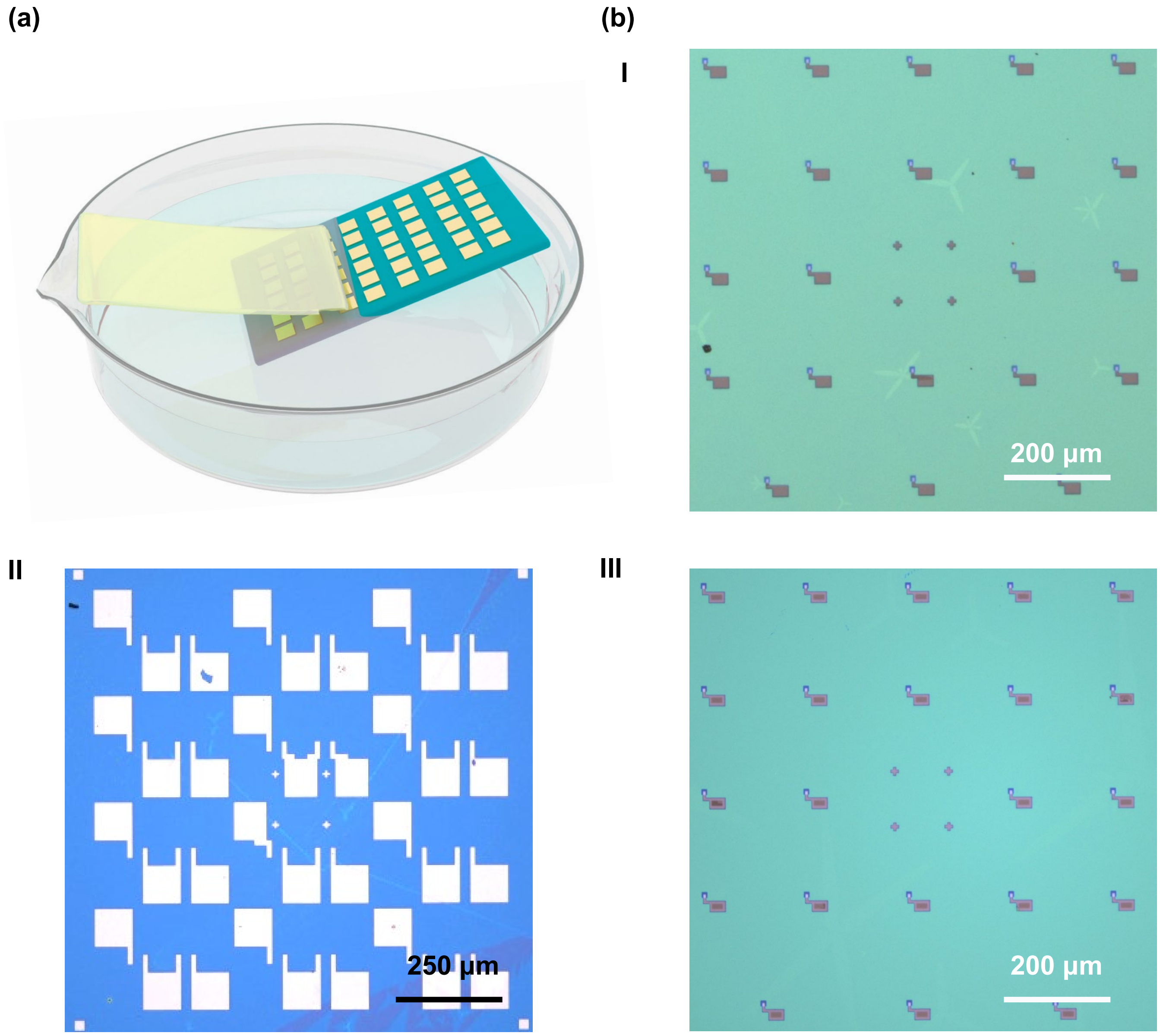

Fig. S3. Low-temperature, damage-free transfer technology of monolayer MoS_2 films. (a) Schematic illustration of the low-temperature, damage-free transfer process of monolayer MoS_2 to a target substrate, utilizing the surface tension of deionized water and the extremely weak adhesion between the monolayer MoS_2 and the substrate. (b) I. Optical microscope image of the transferred sample on the HfO_2 substrate with patterned electrodes. II. Optical microscope image of the transferred sample on the SiO_2 substrate with patterned electrodes. III. Optical microscope image of the patterned MoS_2 channel on an HfO_2 substrate surface.

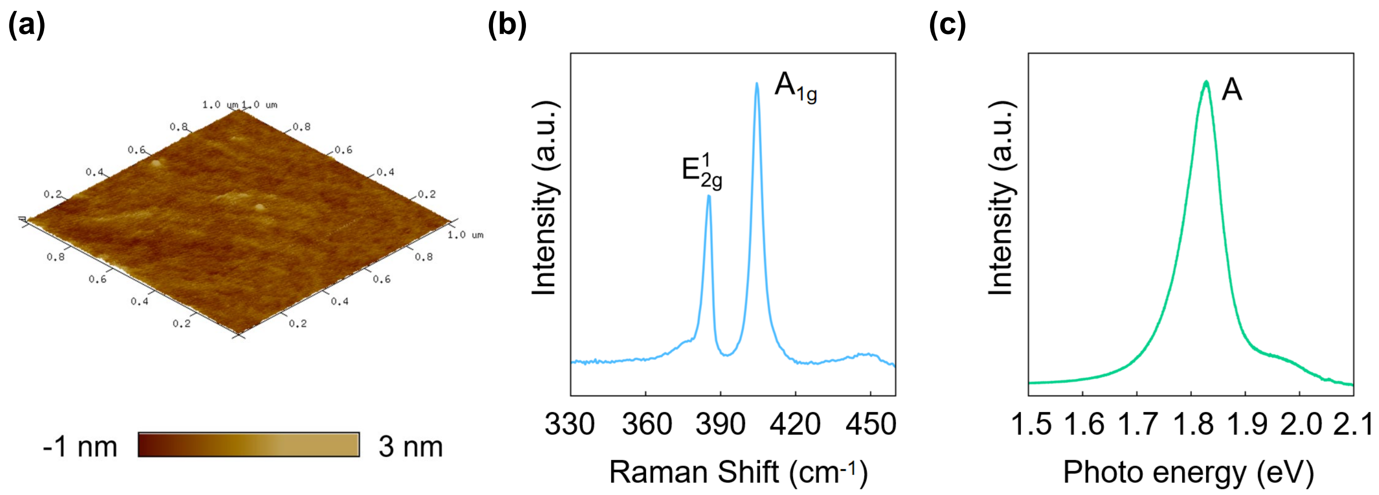

Fig. S4. Characterization of morphology and structure of monolayer MoS_2 films. (a) Atomic force microscopy (AFM) characterization of the surface roughness of monolayer MoS_2 film transferred to the substrate without damage. Scale: 5 µm. Root-mean-square roughness (R_q) = 0.357 nm, and arithmetic mean roughness (R_a) = 0.277 nm. (b) Raman spectroscopy characterization of the monolayer MoS_2 film, with a difference of approximately 19.2 cm^{-1} between the two peaks, consistent with the reference value for monolayer MoS_2 (insert reference). (c) PL spectroscopy characterization of the monolayer MoS_2 film, with the A-peak position at 1.83 eV, close to the bandgap value and consistent with the reference value for monolayer molybdenum disulfide.

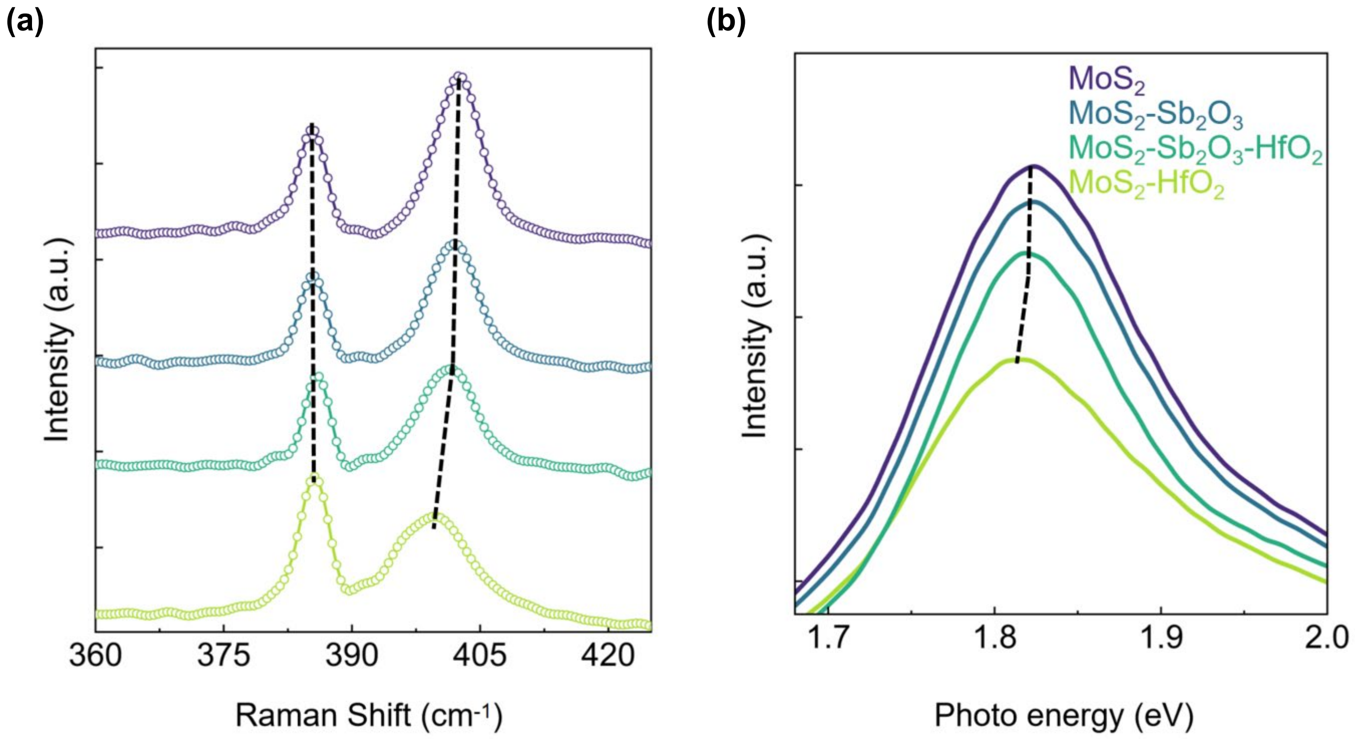

Fig. S5. Spectroscopic characterization of monolayer MoS_2 thin films deposited with different oxide thin films. (a) Raman spectra comparison. (b) PL spectra comparison.

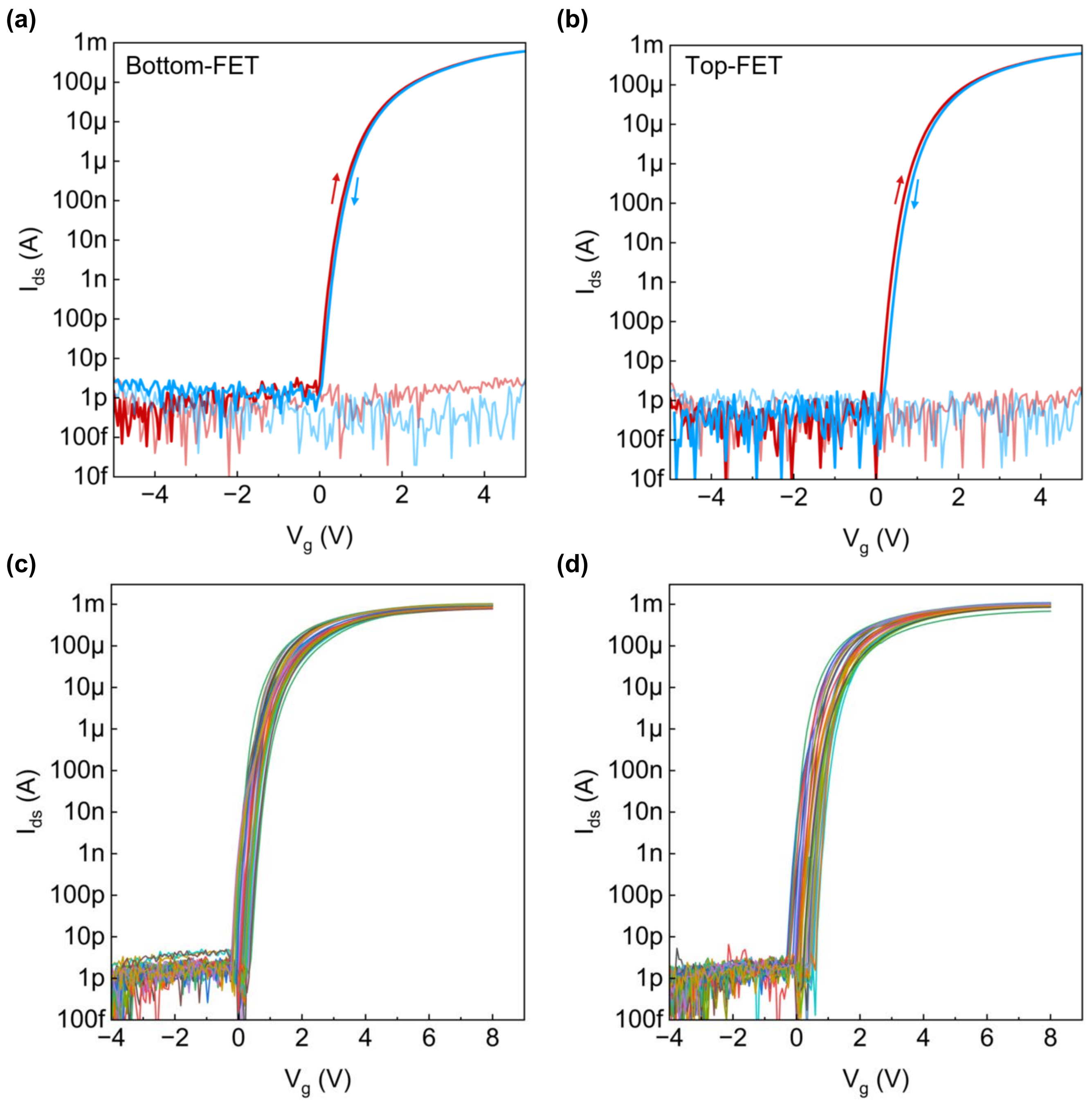

Fig. S6. Comparative analysis of 2-channel monolayer MoS_2 GAA FETs. (a, c) Transfer characteristics and array statistics of the lower channel. (b, d) Transfer characteristics and array statistics of the upper channel. Both layers exhibit voltage hysteresis <100 mV under ±5 V gate sweeps (V_{ds} = 1 V, N = 30 devices). The above FET demonstrates an approximately 50 mV hysteresis window enlargement compared to the bottom FET.

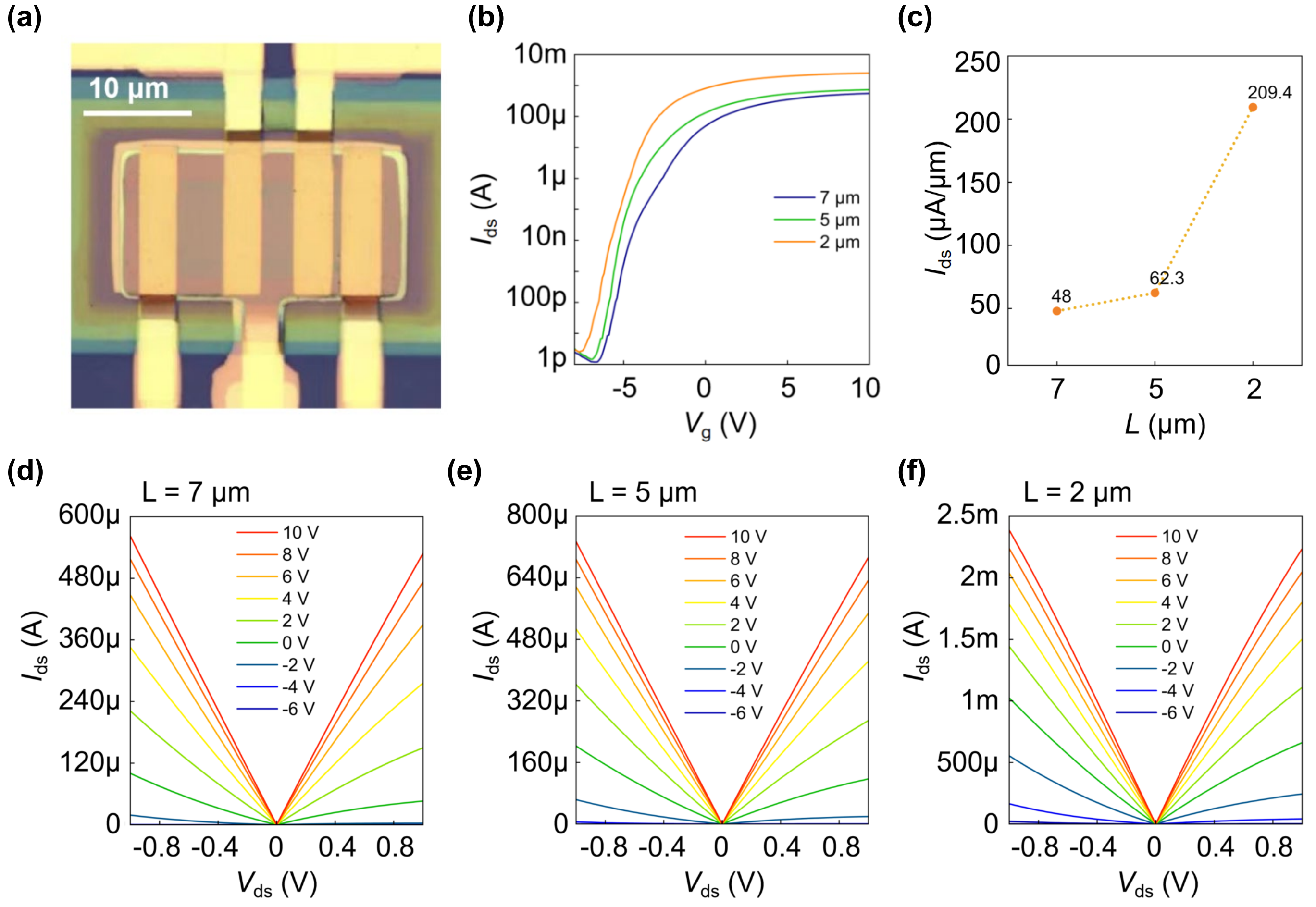

Fig. S7. Comparison of the electrical transport properties of 3D GAA FETs with variable channel lengths via seedless theory. (a) Optical microscope image of the variable-channel GAA FET. (b) Comparison of transfer characteristics curves for GAA FETs with three different channel lengths, with a drain voltage, V_d:1 V. (c) Comparison of current density for GAA FETs with three different channel lengths at a specific gate voltage, V_g:10 V. (d-f) Comparison of output characteristics curves for GAAFETs with three different channel lengths, with a gate voltage scan range of -6 to 10 V and a step of 2 V.

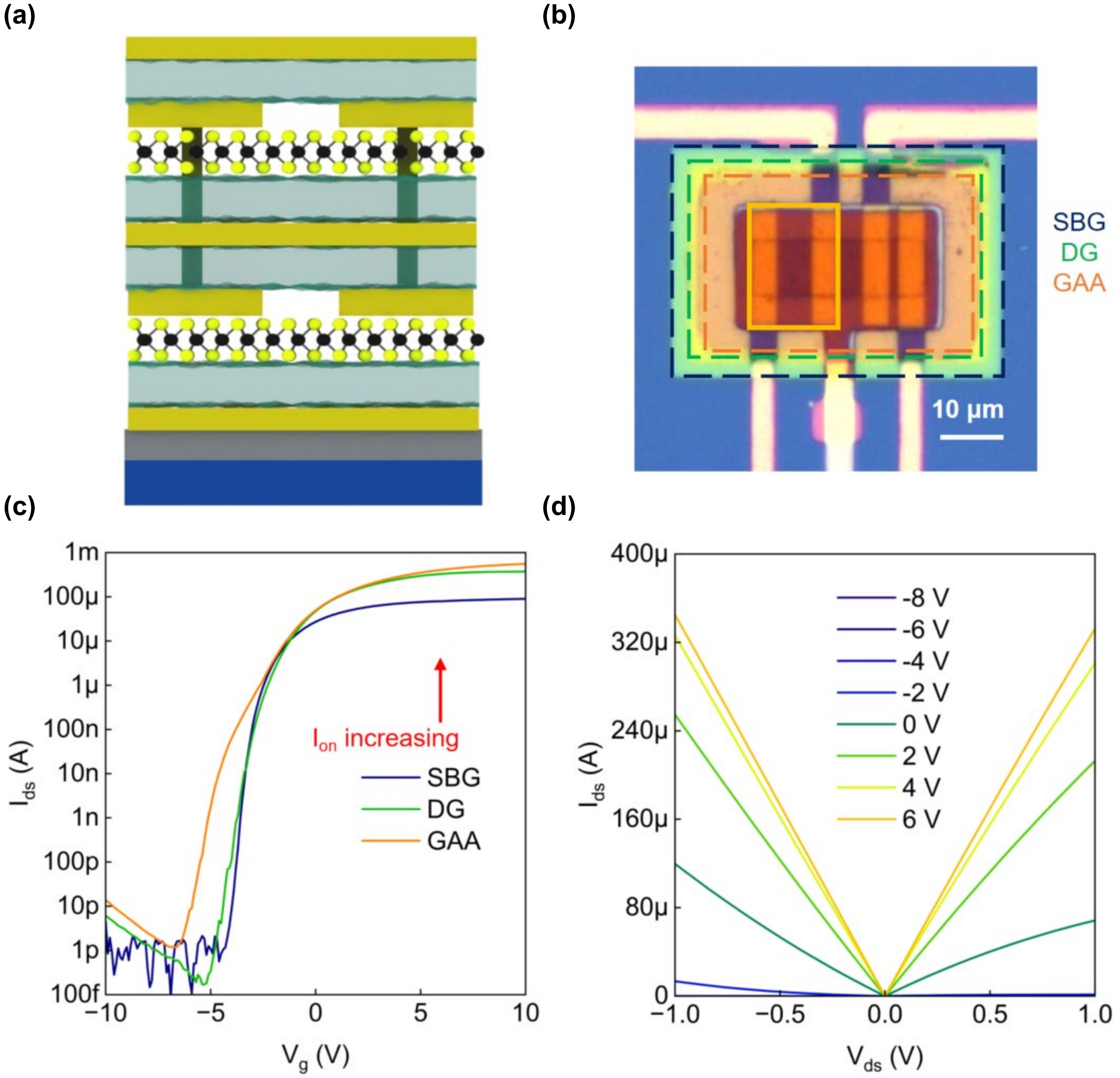

Fig. S8. Comparison of electrical transport properties among three different gate control configurations via seedless theory. (a) Schematic illustration of the device interfaces. (b) Optical microscope image of the device with a width-to-length ratio of 10/7 μm. (c) Transfer characteristic curves of devices with the three different gate control structures. (d) Corresponding output characteristic curves.

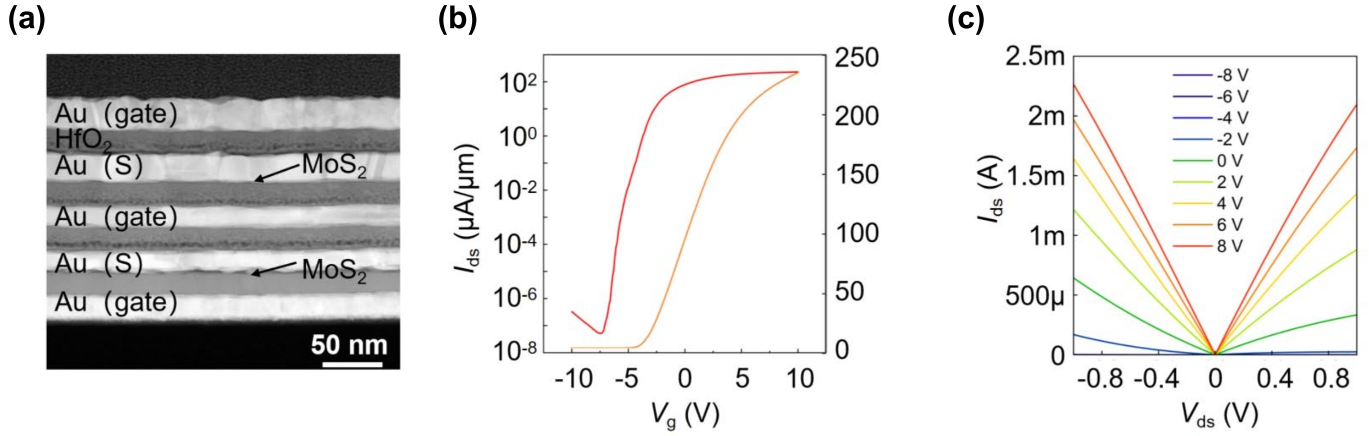

Fig. S9. The 2-channel monolayer MoS_2 GAA FET device fabricated via seedless theory. (a) TEM image of the device with a width-to-length ratio of 8/2 μm. (b) Transfer characteristic curves of the device. (c) Corresponding output characteristic curves.

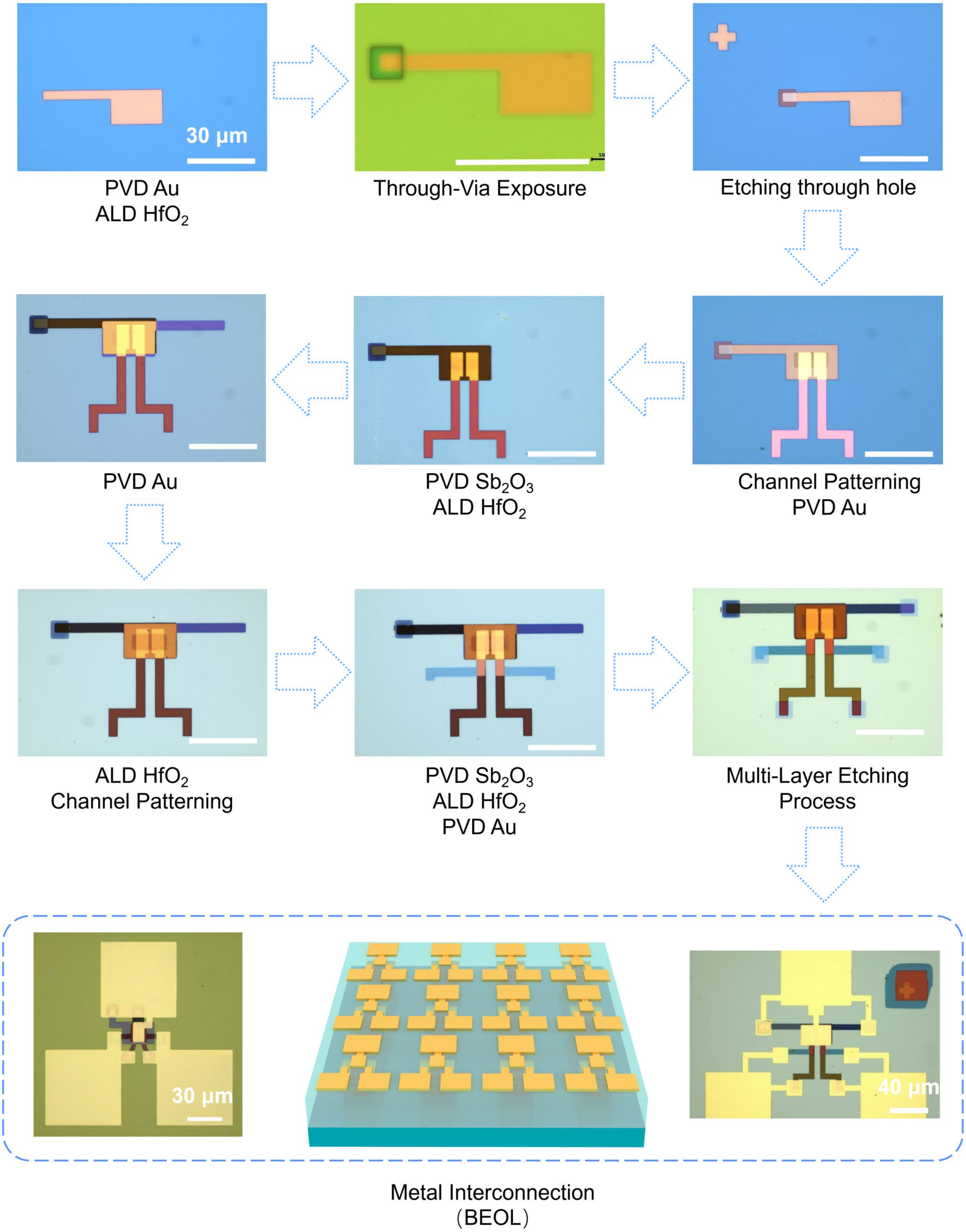

Fig. S10. Critical fabrication steps during large-scale 3D integration of MoS_2 GAA FETs.

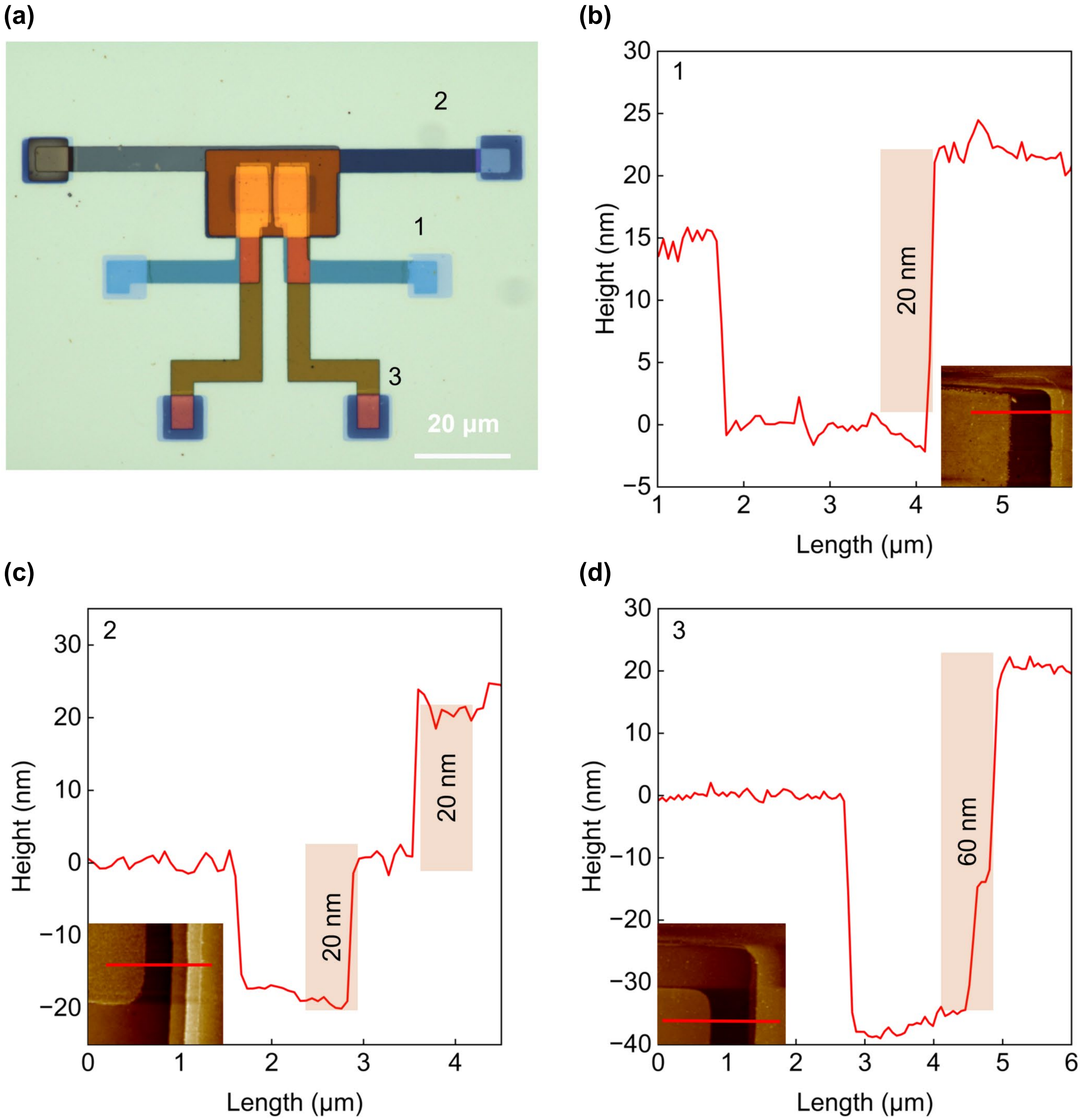

Fig. S11. AFM topography characterization of vias at different layers of monolayer MoS_2 GAA FET devices. (a) Optical microscope image of the device (awaiting subsequent metal interconnection processes). (b-d) AFM surface characterization results of the through-vias after the first to third etching processes of the high-κ dielectric layer. It can be observed that under the same etching parameters, the surfaces of the through-vias remain relatively flat and the depth increases by approximately 20 nm with each successive etching step.

The results also indicate that the etching effect on the gold electrodes is stronger. This is attributed to the fact that during the ALD deposition process, the oxide surface has a stronger affinity for Hf atoms compared to Au, resulting in a denser film formation.

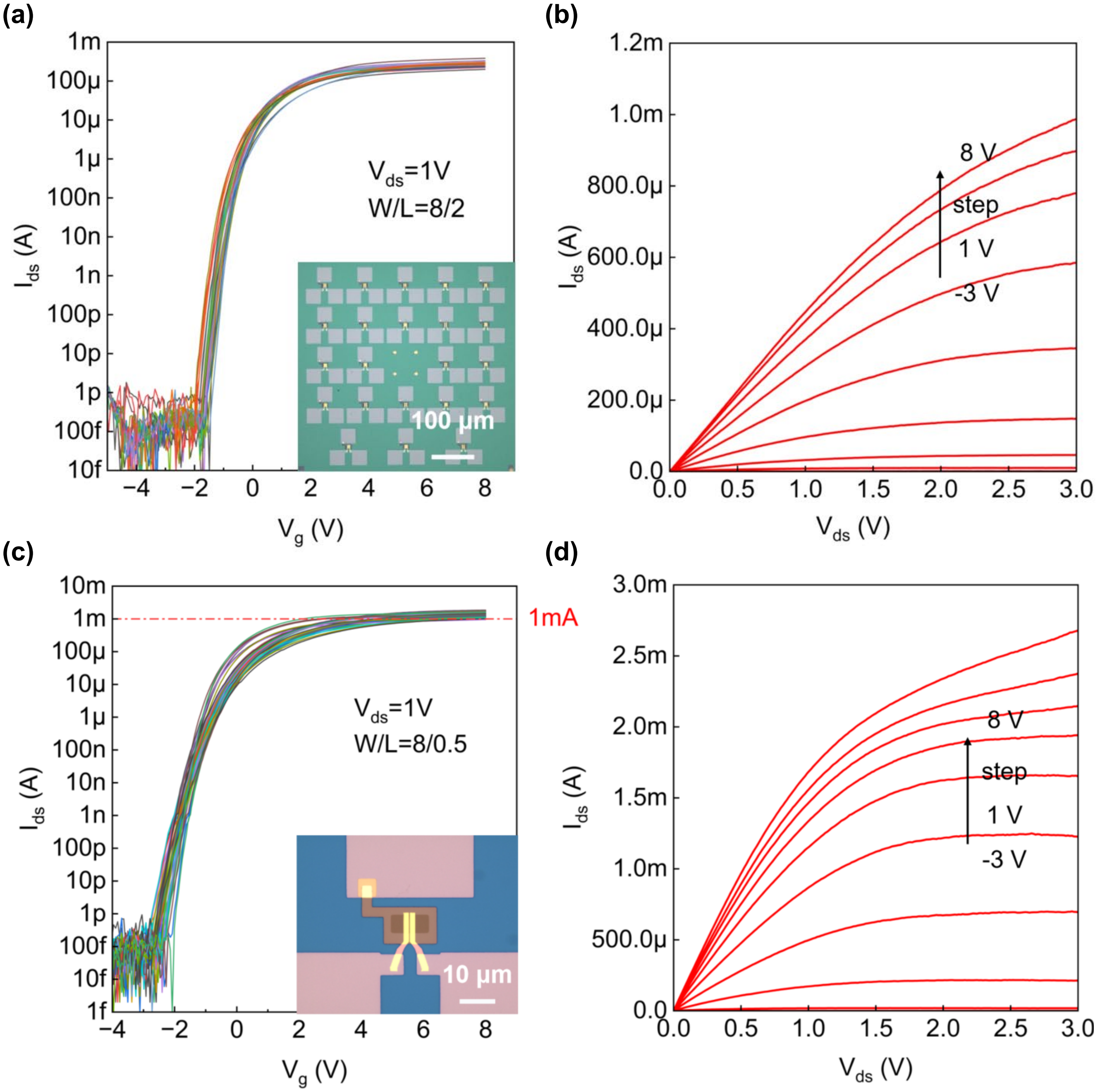

Fig. S12. Performance statistical analysis in back-gated monolayer MoS_2 MOSFET arrays. (a) Statistical distribution of transfer characteristics for a back-gated monolayer MoS_2 MOSFET array (V_{ds} = 1 V, W/L=8/2 μm, N = 25 devices). (b) Representative output characteristics of a single device extracted from the array. (c) Transfer curve statistics for a scaled short-channel array with identical architecture (L_{ch} = 500 nm, N = 53 devices). (d) Output characteristics of a representative short-channel device.

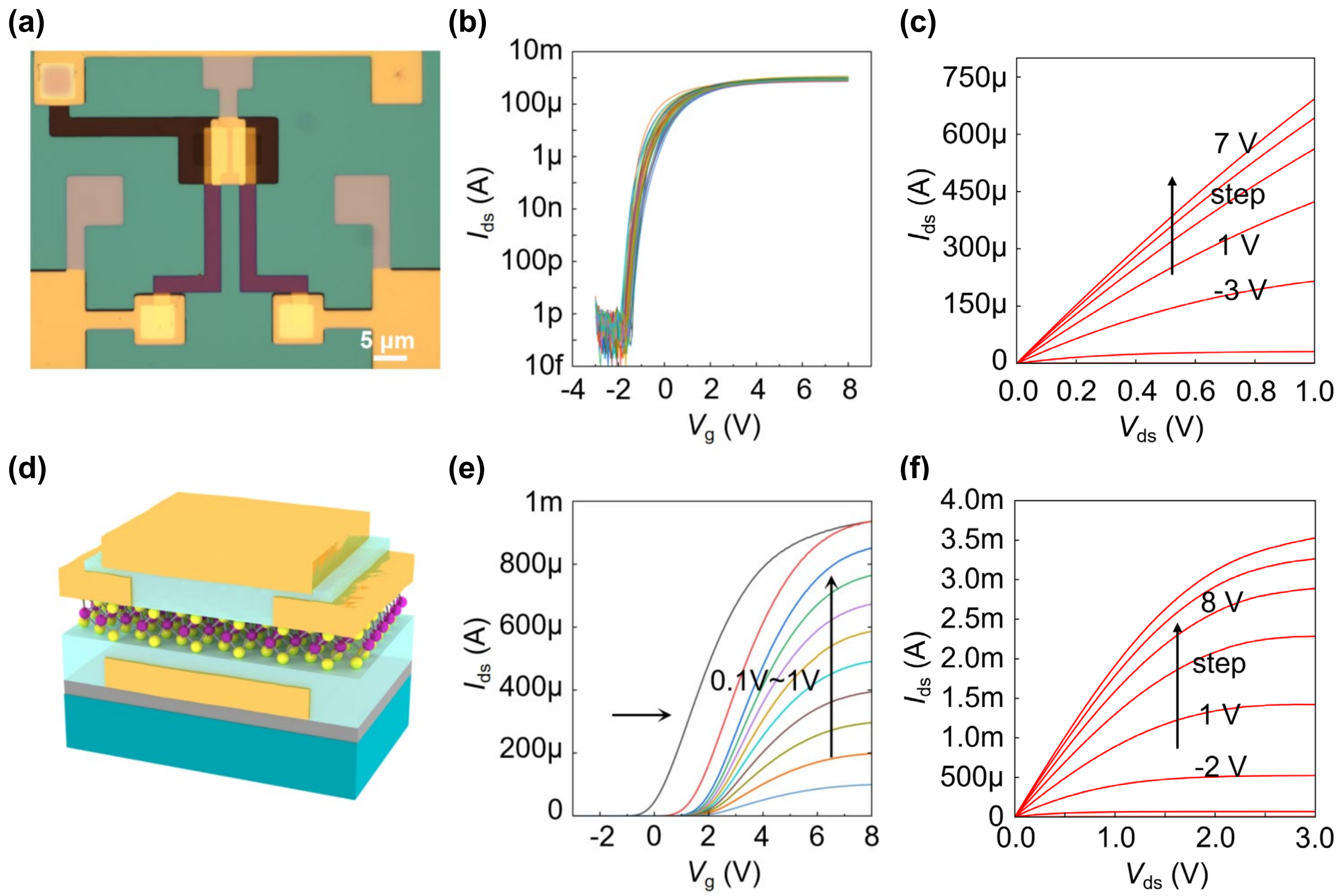

Fig. S13. Performance statistical analysis in 1-channel monolayer MoS_2 GAA FET arrays. (a) Optical micrograph of a single device (scale bar: 5 μm). (b) Statistical distribution of transfer characteristics for the dual-gated array ($N = 57$ devices). (c) Output characteristics of a representative single device. (d) Schematic illustration of the device architecture. (e) Transfer characteristics under varying drain biases (V_{ds} = 0.1-1 V, step: 0.1 V). The device exhibits exceptional switching performance with an ON/OFF ratio exceeding 10^9 at V_{ds} = 100 mV. (f) Output characteristics of a 500 nm channel-length 1-channel monolayer MoS_2 GAA FET.

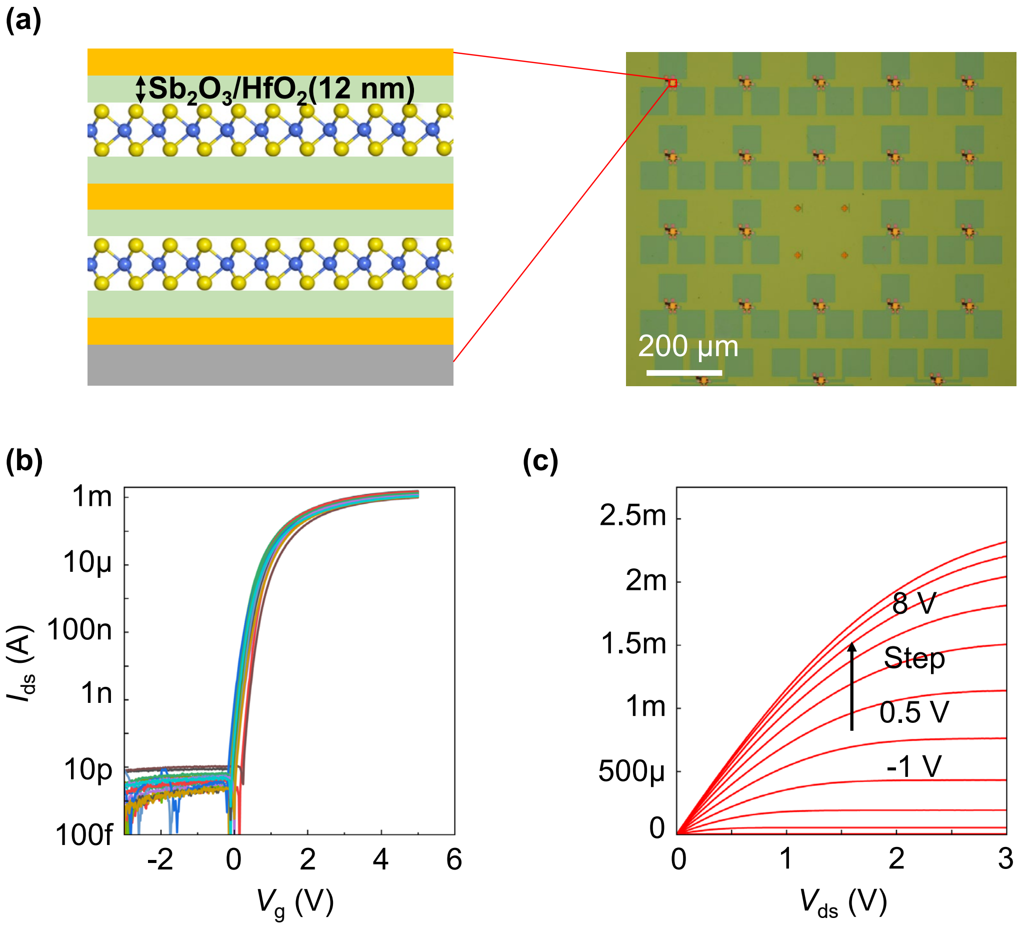

Fig. S14. Performance statistical analysis in 2-channel monolayer MoS_2 GAA FET arrays with thinner gate oxide. (a) Schematic diagram and array optical microscope image of the 2-channel monolayer MoS_2 GAA FET with a thinned dielectric layer (W/L:8/2 μm, T_{ox}=12 nm). (b) Statistical distribution of transfer characteristics for the array (N = 19 devices, V_{ds}=1 V). (c) Output characteristics of this device.

Table S1. The developed material parameter library for MoS_2 and a partial display of the device model parameters constructed based on this library.

Parameters	
μ ($cm^2V^{-1}s^{-1}$)	50
E_g (eV)	1.83
Epsilon	3.93
L_{ch} (μm)	2
Temperature (K)	300
Trap density (cm^{-3})	$2.9e^{12}$
Fix Charge (cm^{-2})	$1\sim2e^{12}$
Carrier Transport Models	PhuMob<br>Coulomb2D
Recombination	Shockley-Read-Hall<br>Auger
Solver	ParDiso

This table presents some of the physics-based calibration model parameters of our TCAD model, including electron mobility, bias voltage, dielectric constant, bandgap width, defect density, fixed charge density, and various structural dimensions. These parameters were derived from literature reports (NE, IEEE, NM) and experimental approximations to enhance the authenticity of the device model.
